# Predicting childhood and adolescent attention-deficit/hyperactivity disorder onset: a nationwide deep learning approach

**DOI:** 10.1038/s41380-022-01918-8

**Published:** 2022-12-19

**Authors:** Miguel Garcia-Argibay, Yanli Zhang-James, Samuele Cortese, Paul Lichtenstein, Henrik Larsson, Stephen V. Faraone

**Affiliations:** 1 School of Medical Sciences, Faculty of Medicine and Health, Örebro University, Örebro, Sweden; 2 Department of Medical Epidemiology and Biostatistics, Karolinska Institutet, Stockholm, Sweden; 3 Departments of Psychiatry and of Neuroscience and Physiology, SUNY Upstate Medical University, Syracuse, NY USA; 4 School of Psychology, University of Southampton, Southampton, UK; 5 Clinical and Experimental Sciences (CNS and Psychiatry), Faculty of Medicine, University of Southampton, Southampton, UK; 6 Solent NHS Trust, Southampton, UK; 7 Hassenfeld Children’s Hospital at NYU Langone, New York University Child Study Center, New York City, New York, NY USA; 8 Division of Psychiatry and Applied Psychology, School of Medicine, University of Nottingham, Nottingham, UK

**Keywords:** ADHD, Psychiatric disorders

## Abstract

Attention-deficit/hyperactivity disorder (ADHD) is a heterogeneous disorder with a high degree of psychiatric and physical comorbidity, which complicates its diagnosis in childhood and adolescence. We analyzed registry data from 238,696 persons born and living in Sweden between 1995 and 1999. Several machine learning techniques were used to assess the ability of registry data to inform the diagnosis of ADHD in childhood and adolescence: logistic regression, random Forest, gradient boosting, XGBoost, penalized logistic regression, deep neural network (DNN), and ensemble models. The best fitting model was the DNN, achieving an area under the receiver operating characteristic curve of 0.75, 95% CI (0.74–0.76) and balanced accuracy of 0.69. At the 0.45 probability threshold, sensitivity was 71.66% and specificity was 65.0%. There was an overall agreement in the feature importance among all models (τ > .5). The top 5 features contributing to classification were having a parent with criminal convictions, male sex, having a relative with ADHD, number of academic subjects failed, and speech/learning disabilities. A DNN model predicting childhood and adolescent ADHD trained exclusively on Swedish register data achieved good discrimination. If replicated and validated in an external sample, and proven to be cost-effective, this model could be used to alert clinicians to individuals who ought to be screened for ADHD and to aid clinicians’ decision-making with the goal of decreasing misdiagnoses. Further research is needed to validate results in different populations and to incorporate new predictors.

## Introduction

Attention-deficit/hyperactivity disorder (ADHD) is a heterogeneous neurodevelopmental disorder characterized by impairing levels of inattention, hyperactivity/impulsivity, or both, with an estimated worldwide prevalence of 5–10% in children and 2–5% in adults [1]. The disorder, which frequently co-occurs with other psychiatric and medical conditions [2–6], among others, leads to economic and interpersonal problems, academic impairments, delinquency, and injuries [4, 7–10] that are associated with a significant individual and healthcare burden [11]. An extensive body of work shows that pharmacologic treatment for ADHD protects against a wide range of adverse outcomes [10] (e.g., injuries and accidents, criminality, substance use disorders, suicide, and traumatic brain injury), at least in the short term.

The substantial heterogeneity and comorbidity of ADHD pose diagnostic challenges for clinicians and can lead to either missed or false positive diagnoses [12, 13]. Missed diagnoses expose patients to the adverse outcomes of the disorder; false positive diagnoses expose patients to improper treatments and their side effects. Delays in correct diagnosis and treatment ultimately engender increased healthcare use, potentially driven by the deterioration of other co-occurrent psychiatric and somatic conditions [11, 14]. Others have sought to address misclassification using objective measures such as genetics [15], blood biomarkers [16], rating scales [17], eye vergence [18], fMRI, EEG, and MRI to classify individuals with and without ADHD [19–22]. This work has not yet led to a method that is routinely used in clinical practice.

Although traditional statistical methods can assess predictive accuracy, they cannot deal with complex non-linear relationships, especially when many predictive features interact with one another to predict outcomes. In contrast, machine learning can handle such complex problems if a sufficiently large sample is available [23]. Although machine learning has been applied to objective data, such applications are limited due to the expense of objective data which limits sample size. Moreover, these small samples sizes are at risk for overestimating accuracy when machine learning methods are not correctly applied [21, 24, 25]. A convenient alternative to testing is the use of register-based data. This approach has previously been used for different outcomes with good discrimination results [26, 27]. It has two main advantages: large samples are available for model estimation and, when models are implemented, there are no costs for collecting data for clinical implementation. Nevertheless, to date there are no studies that applied machine learning techniques to the classification of ADHD using socio-demographic and clinical features from population-based registry data.

In this paper, we aimed to train different machine learning and deep learning algorithms for classifying childhood and adolescent ADHD to 1) aid clinicians’ decision-making in terms of diagnosis, and 2) offer a model for risk stratification and clinical referral of high-risk individuals. All models used Swedish population registry data including variables such as perinatal risk factors, medical and psychiatric comorbidities for the individual and the relatives, criminal convictions for the individual or the biological parents.

## Materials and methods

### Study population

This population-based study used several Swedish registers: the total population register, the medical birth register, the prescribed drug register (PDR), the national patient register (NPR), the multi-generation register, and the national crime register. The total population register includes demographic information for all individuals with permanent residence in Sweden [28]. The medical birth register is a nationwide register with a 99% coverage that contains obstetric information of all deliveries in Sweden [29]. The PDR includes complete information on all dispensed drugs in Sweden from 2005 onwards. The NPR includes medical records from inpatient and outpatient visits since 1973 and 2001, respectively. The multi-generation register consists of family information for all individuals residing in Sweden, and lastly, the national crime register contains all criminal offenses in Sweden for all individuals from the age of criminal responsibility (i.e., 15 years) or older. Our cohort comprised 238,696 individuals born and living in Sweden between 1995 and 1999 with information on their biological parents and who did not emigrate or die before 2013.

### Outcome

Individuals with ADHD were identified based on either the presence of a diagnosis in the NPR (including inpatient and outpatient care services) from age 3 onwards using the International Classification of Diseases (ICD) version 9 code 314, ICD10-code F90 or a recorded prescription of any ADHD medications (Anatomical Therapeutic Chemical [ATC] codes N06BA04, N06BA01, N06BA02, N06BA09, and N06BA12) from the PDR. The outcome variable was dichotomized indicating presence or absence of an ADHD diagnosis (1/0) at any point between 1995 and 2013.

### Features

In order to predict childhood and adolescent ADHD, we considered a set of well-stablished predictors [3, 30–32] based on the availability and quality of this information in the Swedish national registers. Only features with less than 10% missingness were included. Other predictors were not selected because, as recommended in recent guidance [33], we used predictors with existing evidence from prior research and clinical knowledge, and to reduce data dimensionality. We dichotomized and included the following predictors defined as the presence in the NPR of any of the following psychiatric and somatic disorders (for those with ADHD, all predictors should be either before or coincident with the diagnosis of ADHD, and for those without ADHD, it would be prior to age 18): substance use disorder (SUD), major depressive disorder, anxiety disorder, autism spectrum disorder (ASD), obesity, intellectual disability, speech/language developmental disorder and learning disorder, motor and tic disorders, other neurodevelopmental disorders not specified, eating disorder, gastro-esophageal reflux disease, asthma, sleep disorder, hypertension, unintentional injuries, traumatic brain injury, bipolar disorder, allergic rhinitis and allergic conjunctivitis, and allergic dermatitis. We also included: sex, head circumference and weight at birth, small size for gestational age, Apgar score, number of failed subjects at school at age 16 (coded as 0 if the ADHD diagnosis happens before age 16), and presence of criminal convictions. ICD codes used to define all features are presented in Supplementary Table S2.

Amongst the predictors related to the biological parents, we included: Maternal tobacco use during pregnancy, BMI from mother at the first prenatal visit, pregnancy length, type of delivery (vaginal delivery with or without assistance, planned caesarian delivery, or intrapartum caesarian delivery), presence of any criminal convictions of any of the parents. In terms of parental psychiatric disorders, we included all the following if they occurred before or during follow up: ADHD, alcohol use disorder (AUD), SUD, anxiety, eating disorder, depression, bipolar disorder, schizophrenia, personality disorder. In total, 40 features were selected.

### Statistical analysis

**Fig. 1:**
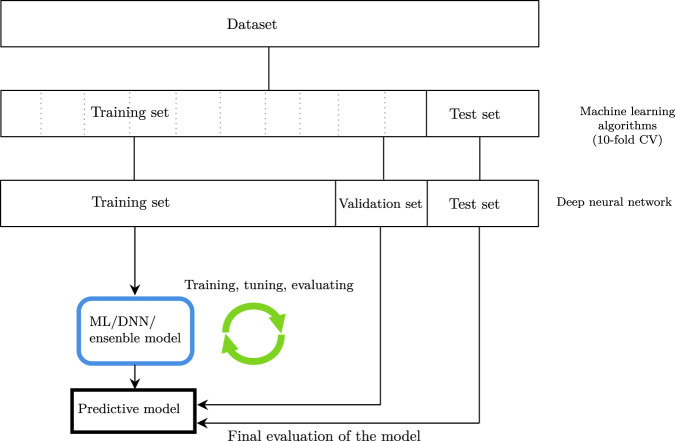
Flowchart of the data split, hyperparameter tuning, and evaluation process. CV cross-validation, ML machine learning, DNN deep neural network.

For all features, zero-variance and near-zero-variance were checked and removed. Highly correlated features (≥0.95) were handled by randomly keeping one of them. Near-zero-variance features were removed to avoid the possibility for those features to become zero-variance during the data splits or cross-validation (CV). Next, an initial 80% stratified data split was performed to be used as the training data. The remaining 20% was used as testing data. For the deep neural network (DNN), the aforementioned 80% split was further split to create a hold-out validation/development set comprising 18% of the training set, and the remaining 82% was used as the training set. As such, all models were evaluated using the same independent testing set, however, to alleviate computational burden when using *k*-fold CV in the training of the DNN, we used a hold-out validation/development set for hyperparameter tuning. A flowchart describing the data split strategy is shown in Fig. 1. Categorical variables with more than three levels were one-hot encoded and categorical variables with two levels were used as binary variables (0/1), and all continuous variables were normalized after the split to avoid information leakage, fitted in the training set, and applied to the training, validation, and test sets. Class imbalance of the outcome in the training set was dealt with using the borderline synthetic minority oversampling technique (SMOTE-1) [34] using 5 nearest neighbors.

We used several machine learning algorithms: logistic regression, random forest (RF), gradient boosting (GB), XGBoost, naïve Bayes (NB), and regularized logistic regression (L1L2) and a DNN. Moreover, a soft-voting ensemble model combining the best performing models in the CV/validation set were trained. A soft-voting ensemble model predicts class membership based on the argmax of the sum of predicted probabilities from each model. Learning curves were plotted to assess the adequacy of our sample sizes. For the machine learning algorithms (i.e., all models except the DNN), hyperparameter optimization was performed using a stratified 10-fold CV in the training set by a manual grid search. To find the optimal hyperparameters in the DNN, we used distributed hyperparameter optimization (hyperopt) [35] with 100 evaluations and Bayesian optimization [36] (200 steps of Bayesian optimization and 5 random points to sample the target function). The model that performed best in the validation data set —and smallest difference between training and validation sets— was chosen and then assessed in the test set. Binary cross-entropy was used as loss function. Supplementary Table S3 shows all hyperparameters and the search space for each model.

Owing to the class imbalance in the test set, we used the Area Under the Receiver Operating Characteristic Curve (AUROC) as the evaluation metric with 95% confidence intervals using a fast implementation of the DeLong algorithm [37]. The AUC in the test set was used as our final estimate of the model’s ability to discriminate those with and without ADHD. We included additional metrics such as balanced accuracy, area under the precision-recall curve (AUPRC), sensitivity, specificity, positive predicted power (PPP), and negative predictive value (NPV) using different thresholds. The precision-recall curve was included because is especially relevant for interpreting the clinical value of models. It plots positive predictive power against sensitivity for every threshold on the model’s output probability. Feature importance was estimated based on mean decrease in impurity and mean absolute Shapley additive explanations (SHAP) values for the DNN. The Kendall rank correlation (τb) was calculated between the ranking of the feature importance to assess the level of agreement between different methods together with bootstrapped 95% confidence intervals with 50,000 replications with replacement. SHAP values were used to explain the predicted probability for ADHD at the individual level to increase interpretability for the DNN. Furthermore, SHAP scores were used as an alternative to permutation feature importance to ease the computational burden. The Guidelines for Reporting Machine Learning Investigations in Neuropsychiatry (GREMLIN [25]) and Strengthening the Reporting of Observational studies in Epidemiology (STROBE) guidelines were followed (see Supplementary). Data management was performed using SAS software version 9.4 and statistical analyses using Python 3.8.13 (scikit-learn [38] version 1.1.1, imbalanced-learn [39] version 0.9.1, XGBoost [40] version 1.6.2, and keras [41] version 2.8.0 libraries). Python code and model weights are available at https://github.com/kmlstyle/ADHD-DNN. See Supplementary for code usage.

## Results

Our cohort comprised 238,696 individuals, of whom 12,893 (5.4%) had ADHD. For the machine learning algorithms, the training dataset had 190,956 observations (10,314 [5.4%] with ADHD) and the testing dataset had 47,740 observations, of which 2579 (5.4%) had ADHD. For the DNN, the training set contained 156,583 individuals, 34,373 in the validation set, and the testing dataset 47,740 observations, of whom 5.4% had ADHD. As expected, those with ADHD were more likely to be diagnosed with the majority of the selected mental disorders and medical conditions (Supplementary Table S1), with the biggest difference in ASD, speech/language developmental and learning disorders, and chronic motor or vocal tic disorder. We also found an increased prevalence of all parental psychiatric and somatic disorders in those with ADHD (Supplementary Table S1).

### Model selection

The number of features was reduced from 40 to 22 based on low variance or high intercorrelations (see Methods). The logistic regression model achieved an AUC in the test set of 0.74 (0.73–0.75). After hyperparameter optimization, the RF model achieved an AUC of 0.68, 95% CI (0.67–0.69) in the test set, whereas the XGBoost model achieved an AUC of 0.69, 95% CI (0.68–0.70). Both models displayed signs of overfitting (training set AUC > 0.92). With a slightly better fit, the GB model and elastic net logistic regression achieved an AUC of 0.73, 95% CI (0.71–0.74) and 0.74, 95% CI (0.73–0.75), respectively. The best fitting model to the data was the DNN that achieved an AUC of 0.75, 95% CI (0.74–0.76) and balanced accuracy of 0.68 in the test set (Table 1).

**Table 1:** AUC with 95% CI in the training and testing sets for the different trained models.

	AUC			
Model	Training	Testing	Balanced accuracy	AUPRC
Logistic regression	0.819	0.742 (0.732–0.753)	0.673	0.162
Random forest	0.930	0.678 (0.667–0.689)	0.620	0.189
Gradient boosting (GB)	0.874	0.726 (0.715–0.737)	0.663	0.177
XGBoost	0.925	0.688 (0.676–0.699)	0.632	0.209
Naïve Bayes (NB)	0.806	0.710 (0.698–0.721)	0.655	0.179
Logistic regression – L1 and L2 penalty (elasticnet)	0.816	0.745 (0.735–0.755)	0.675	0.179
Deep neural network (DNN)	**0.800**	**0.753 (0.743–0.763)**	**0.684**	**0.218**
Ensemble (XGB, GB, NB, L1L2)	0.887	0.743 (0.732–0.752)	0.667	0.208
Ensemble (XGB, GB, NB, DNN)	0.898	0.750 (0.739–0.760)	0.671	0.212

*AUC* Area Under the Receiver Operating Characteristic Curve, *AUPRC* area under the precision-recall curve.

Bold values represent the best performing model for each metric.

**Fig. 2:**
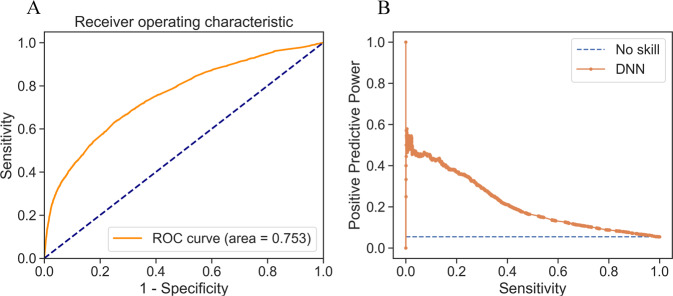
Performance of the deep neural network (DNN). **A** ROC curve **B** Precision-recall plot.

**Fig. 3:**
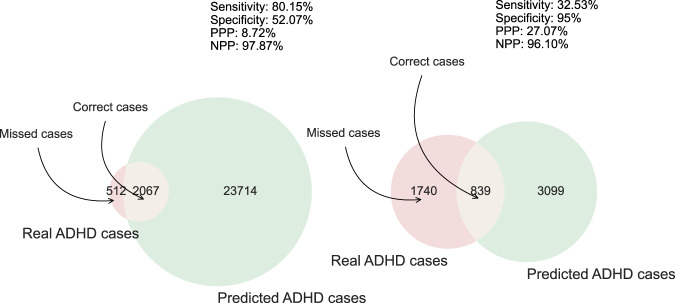
Depiction of the DNN performance predicting ADHD at two different thresholds. PPP Positive predictive power, NPP Negative predictive power. The probability threshold was 0.34 (left) and 0.78 (right).

The DNN was built with two hidden layers of 10 and 15 units respectively with a rectified linear activation function and a dropout layer in between (dropout rate of 0.217). The first hidden layer included a kernel L1 regularizer with a cost function *λ* = 1e-3. The DNN used the Adadelta optimizer (learning rate 7e-3) to train the model for 200 epochs with a batch size of 40 with the Xavier normal weight initializer (Glorot). Supplementary Fig. S1 depicts the learning history with respect to the loss function and AUC. The good convergence for both metrics between the training and validation sets does not provide evidence of overfitting. Learning curves showed a learning plateau on around 260,000 samples. This pattern indicates that increasing our sample size will not improve accuracy (see supplementary Fig. S2). Instead, to improve accuracy we need to add more features or improve the model’s capacity for learning. Each model computes for each person a probability of being diagnosed with ADHD. By choosing a threshold on the output probability, we sort persons into those predicted to have ADHD and those predicted not to have ADHD. Supplementary Table S4 presents sensitivity, specificity, PPP, and NPP at different output probability thresholds, and Fig. 2 presents the precision-recall and receiver operating characteristic curves. The precision-recall curve shows that to achieve a sensitivity of 80% one must accept a PPP below 10% (see Fig. 3 for graphical representation of the model’s performance using two different thresholds).

The top six most important features were having a relative with criminal convictions, sex of patient, having a relative with ADHD, number of academic subjects failed, speech/learning disabilities, and ASD diagnosis. All models showed high agreement as depicted by the correlations (τ > 0.5 for all models, except for the XGB-RF and XGB-DNN comparisons, τ = 0.42 and 0.32, respectively). The complete list of feature importance for the RF, GB, XGBoost, and DNN algorithms together with pairwise Kendall’s τ correlations are shown in Table 2.

**Table 2:** Ranked feature importance for the XGBoost, gradient boosting (GB), random forest (RF), and deep neural network (DNN) models.

Feature	XGB	GB	RF	DNNª	Average
Criminal conviction of either parent	**18**	**20**	**19**	**20**	19.3
Sex	15	**19**	**18**	**21**	18.3
ADHD relative	**21**	15	15	**18**	17.3
Number of academic subjects failed	9	**21**	**20**	**19**	17.3
Speech/learning disability	**20**	**18**	17	10	16.3
Autism disorder	**19**	16	16	12	15.8
Depression	16	14	13	16	14.8
Depression relative	13	17	14	15	14.8
Head circumference	3	13	**21**	13	12.5
Alcohol disorder relative	14	12	12	6	11.0
Anxiety	11	11	11	9	10.5
Criminal conviction	10	8	6	17	10.3
Motor/tic disorders	17	10	8	0	8.8
Allergic rhinitis and Allergic conjunctivitis	7	6	5	14	8.0
Asthma relative	8	9	10	4	7.8
Sleep disorders	12	7	4	8	7.8
Anxiety relative	2	5	9	11	6.8
Allergic dermatitis	6	2	3	7	4.5
Substance use disorders relative	0	3	7	5	3.8
Eating disorders	5	4	1	3	3.3
Small size for age	4	1	2	2	2.3
Eating disorders relative	1	0	0	1	0.5
*Kendall’s τb*	*XGB*	*GB*	*RF*	*DNN*	*Average*
XGB	1	-	-	-	-
GB	0.56^***^ (0.32–0.76)	1	-	-	-
RF	0.42^**^ (0.11–0.70)	0.81^***^ (0.64–0.95)	1	-	-
DNN	0.32^*^ (0.02–0.58)	0.55^***^ (0.30–0.75)	0.52^***^ (0.27–0.72)	1	-
Average	0.62^***^ (0.39–0.81)	0.88^***^ (0.77–0.96)	0.78^***^ (0.60–0.92)	0.65^***^ (0.42–0.85)	1

Importance ranging from 0 (less important) to 21 (most important). Confidence intervals based on 50,000 bootstrap replicates. ^ª^Rank based on SHAP feature importance (mean absolute Shapley values).

^*^p < 0.05, ^**^p < 0.01, ^***^p < 0.001.

Bold values represent the best performing model for each metric.

SHAP values increase the interpretability of DNNs by explaining the predicted probability for ADHD of each individual. Supplementary Fig. S3 shows three individuals with low, medium, and high risk for ADHD. The average predicted probability was 42%. The first individual has a low predicted risk for ADHD of 11%. The risk is low given that this individual is female, no records of ADHD, depression, or criminal convictions for any of the parents, no clinical records of depression, and no academic subjects failed. In contrast, the second individual is a male with a parent with criminal convictions that substantially increases the risk of ADHD. The last individual has been predicted with a high probability of ADHD due to risk-increasing features such as ASD, being male, having a relative with anxiety disorders, allergic rhinitis and allergic conjunctivitis, and allergic dermatitis (features with SHAP values <0.005 are not shown).

## Discussion

We trained machine-learning algorithms to classify childhood and adolescent ADHD using registry data. This is the first study combining Swedish national registry data and machine learning/deep learning techniques to assess the combined accuracy of 22 predictors, including psychiatric and somatic comorbidities, criminal convictions, perinatal variables, and parental psychiatric comorbidities, to predict the onset of ADHD. The best-fitting model was a DNN. It achieved good discrimination in an independent test set (AUC = 0.75, 95% CI [0.74–0.76]). The DNN outperformed all other algorithms in all metrics.

Although the DNN AUC of 0.75 is modest, several considerations suggest that the DNN would be clinically useful. As Ross et al. [42]. have shown, the clinical utility of a model depends on the relative costs of using or not using the model in clinical practice. For our model, deployment costs are limited to the programming required to implement it in the electronic health record. The only other implementation cost is the clinical action required when the model flag a patient as potentially having ADHD. This can be very low, for example, if the patient or parent is asked to complete a rating scale for ADHD symptoms and those results are used determine of a full clinical work up is needed. Not implementing the model would have high costs due to failure to appropriately diagnose and treat ADHD (for a summary of cost studies, see Faraone et al. [3]). Because the model can also alert the clinician that a patient they think may have ADHD may not have ADHD, it could avoid the costs associated with over diagnosing ADHD and may be a useful signal of potential malingering, e.g., by patients who plan to divert or misuse medications [43].

In practice, the model could be applied by selecting a meaningful cut point on the model’s predicted probability that an individual has ADHD. For example, Fig. 2B shows that the PPP increases its rate of decline at a sensitivity of 33% and a PPP of 27%. Using that cut point, our model would correctly identify one third of individuals with ADHD, and among those predicted to have ADHD, 27% would have ADHD. In a hypothetical sample of 1 million individuals (assuming a prevalence of 5.3% for ADHD), our model would detect 17,561 cases out of 54,000, and amongst all individuals predicted to be at risk for ADHD, 14,618 would have ADHD.

There was a good agreement in the importance of the top predictors between different models with an average Kendall’s τb correlation > 0.5. The most important predictors for ADHD were having a parent with criminal convictions, sex of patient, having a parent with ADHD, number of academic subjects failed, and speech/learning disabilities. Previous literature had already established these relationships [30–32], but these predictors have rarely been used together for risk prediction models. This approach showed that all models indicated the importance of having a parent with criminal convictions, ADHD, and depression, demonstrating the complex, multifactorial etiology of ADHD, with an interplay of both genetic and environmental factors in its pathogenesis. The fact that having a parent with criminal convictions or depression ranked high suggests the contribution of environmental risk factors, via epigenetics to ADHD pathophysiology. Epigenetics refers to the modification of gene function and the expression of a phenotype through changes in DNA methylation and histone modifications without a change in the underlying DNA sequence. Epigenetics has been suggested to explain complex mechanisms such as gene–environment interactions that can result in different outcomes due to a similar genotype but different environmental factors. In our cohort, these environmental influences caused by a parent with depression or criminal convictions could have occurred during embryonic development, early infancy, or adolescence altering the expression of genes associated with ADHD and, in turn, affecting brain function with subsequent changes in behavior. Consistent with this idea, previous research indicated epigenetic modifications linked to ADHD such as DNA methylation, histone modifications and expression of noncoding micro RNAs (miRNA) [44–49]. Alternatively, the importance given to both parental criminal convictions and depression could be driven by the co-occurrence of ADHD in the parents rather than these two conditions per se, and thus, indirectly predicting the offspring ADHD due to the high heritability of the disorder.

Regarding clinical utility, our top features could potentially alert clinicians for an early assessment using our model. Feature importance was similar for men and women, with the exception of head circumference and criminal convictions, which showed a larger importance in women. Similarly, having depression and a parent with ADHD displayed a higher importance among men (see Table 3). It is important to note that it is plausible that top features might have masked the importance of lower-ranked features that are redundant with higher ranked features. Nevertheless, our model has the potential to facilitate precision medicine by providing individual-level risk predictions and their unique risk-relevant features. Of note, one of the top features contributing to classification was represented by speech/learning disabilities. This is of relevance as generally practitioners in primary, but also secondary care, are not familiar with this disorder. This highlights the need for raising awareness and training on speech/learning disabilities in primary and secondary care.

**Table 3:** Ranked feature importance for the deep neural network (DNN) model.

Rank	Males	Females
21	Criminal conviction of either parent	Criminal conviction of either parent
20	Number of academic subjects failed	Number of academic subjects failed
19	ADHD relative	Head circumference
18	Depression relative	ADHD relative
17	Depression	Depression relative
16	Head circumference	Allergic rhinitis and allergic conjunctivitis
15	Allergic rhinitis and allergic conjunctivitis	Criminal conviction
14	Anxiety	Anxiety relative
13	Anxiety relative	Anxiety
12	Autism disorder	Autism disorder
11	Criminal conviction	Depression
10	Allergic dermatitis	Sleep disorders
9	Sleep disorders	Allergic dermatitis
8	Eating disorders	Alcohol disorder relative
7	Speech/learning disability	Asthma relative
6	Alcohol disorder relative	Speech/learning disability
5	Substance use disorders relative	Substance use disorders relative
4	Asthma relative	Motor/tic disorders
3	Motor/tic disorders	Small size for age
2	Small size for age	Eating disorders relative
1	Eating disorders relative	Eating disorders

Rank based on SHAP feature importance (mean absolute Shapley values). Importance ranging from 1 (less important) to 21 (most important).

With an estimated incremental annual cost of 2476€, individuals who do not get an ADHD diagnosis until mid-adulthood require increased healthcare utilization compared to those without ADHD (2870€ versus 394€) [11], and compared with those who get their ADHD diagnosis in childhood [14]. This difference stresses the importance of an early correct diagnosis and treatment for those with ADHD and points out how current diagnostic methods do not perform adequately in certain cases. For such cases, applying our predictive model in Sweden, even with a relatively low PPP, should ultimately reduce public health costs and shorten impairments associated with ADHD after treatment initiation. The net benefit would most likely be positive, as shown in other prediction interventions with low PPP such as suicide [42]. It is important to note that this model is not meant to replace well-validated assessment tools, but to alert clinicians to patients who ought to be screened for ADHD or to have them do a more detailed assessment of those who might be malingering.

Amongst the strengths from this paper, we can highlight the use of a large, nationwide sample and the longitudinal nature of the study for 18 years. Additionally, in Sweden, ADHD is assessed at outpatient clinics exclusively by specialist psychiatrists after clinical somatic and psychiatric evaluation. An external validation of the diagnoses in the National Inpatient Register showed high validity with PPPs ranging from 85–95% [50]. Despite these strengths, our conclusions should be interpreted in light of some limitations. Due to the computational burden of hyperparameter search in the DNN when performing k-fold CV with a big sample size using a CPU, we used an independent validation set for this purpose. This approach allowed us to reduce computation time by a factor of *k* and increase the maximum number of evaluations in the hyperparameter optimization process by the same *k* factor. However, since we used a different validation method for hyperparameter optimization in the DNN model, comparing its performance to the other methods should be made with caution. Given that this was a registry-based study, ADHD diagnoses only capture clinically referred cases of ADHD. Thus, our model may not be relevant for cases of ADHD in the population that do not seek treatment. There are several important predictors of ADHD that we were not able to include given the unavailability within the registers, including IQ, reading and arithmetic scores, working memory, reaction time, risky decision-making [3] that future studies looking to improve the model performance can incorporate. Further, given that we used well-stablished predictors, we were not able to identify predictors than may not have been studied before. Although our model achieved good results when testing it with data that the model never saw before (i.e., the testing set), this does not necessarily imply that it would perform well in other populations. It is plausible that the magnitude of a feature that has a higher influence on the model’s performance may be much greater in one country than another. For instance, it is possible that a country with a low life expectancy rate or low income would have less features that are strongly correlated with ADHD. By the same token, features such as academic performance and a criminal conviction of a parent might have a much lower importance in countries with a high crime rate and low income. Thus, it is imperative to assess the model’s performance in samples from another country or health care system and to perform an evaluation of feature importance. Also, cross-study variability in feature importance may also reflect biased data (e.g., underdiagnosis of diagnoses or differential misclassification). Our study plan called for only included predictors that had previously been shown to be related to ADHD. Future work could use machine learning methods to discover additional features that might improve model accuracy. Lastly, learning curves indicated that increasing our sample size will not improve accuracy, however, including new features or different types of neural networks could potentially improve the model’s performance.

In conclusion, in this paper we presented a DNN model for discriminating childhood and adolescent ADHD using register-based data. The DNN model presented good discrimination and could potentially improve decision-making.

## Supplementary information

Supplementary material

## Data Availability

The Public Access to Information and Secrecy Act in Sweden prohibits us from making individual level data publicly available. Researchers who are interested in replicating our work can apply for individual level data at Statistics Sweden: www.scb.se/en/services/guidance-for-researchers-and-universities/.
