# Supplementary material for: Predicting childhood and adolescent attention-deficit/hyperactivity disorder onset: a nationwide deep learning approach
Source: Mol Psychiatry. 2022 Dec 19;28(3):1232–9. doi: 10.1038/s41380-022-01918-8 (PMC10005952; doi:10.1038/s41380-022-01918-8)
Supplement: Supplementary file 1 — Supplementary material [file 41380_2022_1918_MOESM1_ESM.docx]

**Supplementary material**

**Predicting childhood and adolescent attention-deficit/hyperactivity disorder onset: a nationwide deep learning approach**

Miguel Garcia-Argibay, Ph.D., Yanli Zhang-James, MD, Ph.D., Samuele Cortese, MD, Ph.D., Paul Lichtenstein, Ph.D., Henrik Larsson, Ph.D., Stephen V. Faraone, Ph.D.

**Contents**

[Table S1. Summary of the features for the cohort and stratified for individuals with and without ADHD. 2](#_Toc116316559)

[Table S2. ICD codes used to define each psychiatric/medical condition 3](#_Toc116316560)

[Table S3. Grid-search parameter space for each trained model. 4](#_Toc116316561)

[Table S4. Sensitivity, specificity, PPP, and NPP at different thresholds 5](#_Toc116316562)

[Fig. S1. Training history (loss and AUC) for the DNN. 6](#_Toc116316563)

[Fig. S2. Learning curves for the DNN models showing model accuracy. 7](#_Toc116316564)

[Fig. S3. SHAP values explaining the predicted probability for ADHD of three different individuals. 8](#_Toc116316565)

[STROBE Statement 9](#_Toc116316566)

[Guidelines for Reporting Machine Learning Investigations in Neuropsychiatry (GREMLIN) 14](#_Toc116316567)

[Code availability and use 22](#_Toc116316568)

# Table S1. Summary of the features for the cohort and stratified for individuals with and without ADHD.

| **Feature** | **Overall** | **No ADHD** | **ADHD** | ***p*-value*^2^*** |
| --- | --- | --- | --- | --- |
|  | **(N = 238,696)*^1^*** | **(N = 225,803)*^1^*** | **(N=12,893)*^1^*** |  |
| Sex |  |  |  | <0.001 |
| Male | 122,308 (51%) | 113,654 (50%) | 8,654 (67%) |  |
| Female | 116,388 (49%) | 112,149 (50%) | 4,239 (33%) |  |
| Head circumference (cm) | 35 (2) | 35 (2) | 35 (2) | 0.017 |
| Small for gestational age | 4,825 (2.0%) | 4,505 (2.0%) | 320 (2.5%) | <0.001 |
| Criminal convictions | 11,879 (5.0%) | 11,345 (5.0%) | 534 (4.1%) | <0.001 |
| Criminal conviction (parent) | 116,066 (49%) | 107,835 (48%) | 8,231 (64%) | <0.001 |
| Number of academic fails age 16 | 0 (2) | 0 (2) | 0 (3) | <0.001 |
| *Psychiatric disorders* |  |  |  |  |
| Depression | 5,650 (2.4%) | 4,630 (2.1%) | 1,020 (7.9%) | <0.001 |
| Anxiety | 6,200 (2.6%) | 5,225 (2.3%) | 975 (7.6%) | <0.001 |
| Autism disorder | 2,441 (1.0%) | 1,589 (0.7%) | 852 (6.6%) | <0.001 |
| Eating disorder | 3,398 (1.4%) | 3,169 (1.4%) | 229 (1.8%) | <0.001 |
| Sleep disorder | 2,765 (1.2%) | 2,259 (1.0%) | 506 (3.9%) | <0.001 |
| Eating disorders (parent) | 1,247 (0.5%) | 1,098 (0.5%) | 149 (1.2%) | <0.001 |
| Anxiety (parent) | 20,751 (8.7%) | 18,400 (8.1%) | 2,351 (18%) | <0.001 |
| Depression (parent) | 25,199 (11%) | 22,440 (9.9%) | 2,759 (21%) | <0.001 |
| SUD (parent) | 9,914 (4.2%) | 8,486 (3.8%) | 1,428 (11%) | <0.001 |
| AUD (parent) | 16,926 (7.1%) | 14,807 (6.6%) | 2,119 (16%) | <0.001 |
| ADHD (parent) | 3,867 (1.6%) | 2,496 (1.1%) | 1,371 (11%) | <0.001 |
| Speech/learning disability | 2,579 (1.1%) | 1,755 (0.8%) | 824 (6.4%) | <0.001 |
| Motor/tic disorders | 1,288 (0.5%) | 853 (0.4%) | 435 (3.4%) | <0.001 |
| *Somatic disorders* |  |  |  |  |
| Allergic rhinitis/allergic conjunctivitis | 17,408 (7.3%) | 16,595 (7.3%) | 813 (6.3%) | <0.001 |
| Allergic dermatitis | 11,150 (4.7%) | 10,570 (4.7%) | 580 (4.5%) | 0.3 |
| Asthma (parent) | 12,933 (5.4%) | 11,874 (5.3%) | 1,059 (8.2%) | <0.001 |

*Note.* ^1^n (%); Median (SD). ^2^Pearson's Chi-squared test; Wilcoxon rank sum test

# **Table S2.** ICD codes used to define each psychiatric/medical condition

| **Variable** | **ICD-10** | **ICD-9 (parental)** |
| --- | --- | --- |
| ADHD | F90 | 314 |
| SUD | F32–34 | 291,292,303-305 |
| Depression | F32–34 | 296B, 300E, 311 |
| Anxiety disorders | F40-F42, F44-F45, F48 | 300A, 300C |
| Autism disorder | F84 | - |
| Obesity | E66 | - |
| Intellectual disability | F70 -F73, F78, F79 | - |
| Unintentional injuries | S0-S9, T0-T78, V, W, X1-X5 | - |
| Speech/language developmental disorders and learning disorders | F80, F81, F83, R48 | - |
| Motor and tic disorders | F82, F98.4, F95 | - |
| Other neurodevelopmental disorders not specified | F88, F89 | - |
| Eating disorders | F50.0-F50.3, F50.9, F98.2 | 307B, 307F |
| Sleep disorders | G47, F51 | - |
| Gastro-esophageal reflux disease | K21, R12, K44, K20 |  |
| Hypertension | I10-I15, or I67.4 | - |
| Traumatic brain injury | S01.0-S02.3, S02.7-S02.9, S04.0, S06.0-S07.1, S07.8-S07.9, S09.7-S09.9, T01.0, T02.0, T04.0, T06.0, T90.1-T90.2, T90.4-T90.5, T90.8-T90.9 | - |
| Bipolar disorder | F30, F31 | 296, 298B |
| Asthma | J45, J46 | 493 |
| Allergic rhinitis and allergic conjunctivitis | J30, H101 | - |
| Allergic dermatitis | L20 | 303, 305A, 291 |
| Alcohol use disorder (relative) | F10 | - |
| Schizophrenia | F20 | 295,297,298 |
| Personality disorder | F60-F62, F69 | 301 |

# Table S3. Grid-search parameter space for each trained model.

| **Model** | **Parameter** | **Search space** | **Increments** | **Best** |
| --- | --- | --- | --- | --- |
| Random forest | n_estimators | 20 50 70 100 200 300 400 | - | 400 |
|  | max_depth | 4 to 50 | 2 | 50 |
|  | min_samples_split | 2 to 10 | 2 | 2 |
|  | min_samples_leaf | 1, 2, 4 | - | 1 |
| Gradient Boosting | Learning_rate | 0.05 to 0.2 | - | 0.1 |
|  | n_estimators | 20 50 70 100 200 300 400 | - | 400 |
|  | max_depth | 4 to 50 | 2 | 20 |
|  | criterion | gini, entropy, log_loss | - | gini |
|  | min_samples_split | 2 to 10 | 2 | 2 |
|  | min_samples_leaf | 1, 2, 4 | - | 1 |
| XGBoost | n_estimators | 20 50 70 100 200 | - | 200 |
|  | reg_lambda | 0 to 10 | - | 0.1 |
|  | gamma | 0 to 10 | - | 0.1 |
|  | learning_rate | 0.05 to 0.2 | - | 0.2 |
|  | max_depth | 1 to 20 | 2 | 20 |
|  | scale_pos_weight | None to 3 | - | 1 |
| Penalized regression | Lambda λ | 0, 0.001, 0.005, 0.01, 0.05, 0.1, 0.5 | - | 0.003 |
|  | L1 ratio | 0 to 1 | 0.2 | 1 |
| Naïve Bayes | var_smoothing | 1e-4 to 1e-10 | - | 1e-5 |
| Deep neural network | Optimizers | Adam, SGD, Adadelta, RMSprop, Adagrad, Adamax, Nadam | - | Adadelta |
|  | Activation functions | Relu, Selu, LeakyReLU, sigmoid, softplus, softsign, tanh, eli, exponential | - | Relu |
|  | Batch size | 5 to 80 | Uniform | 40 |
|  | Dropout rate | 0 to 0.5 | Uniform | 0.217 |
|  | Learning rate | 0 to 0.1 | Uniform | 7e-3 |
|  | Kernel L1 regularizer, λ | 0, 1e-4, 1e-3, 1e-2 | - | 1e-3 |
|  | Number of layers | 1 to 6 | - | 2 |
|  | Number of neurons layer 1 | 5 to 20 | 5 | 10 |
|  | Number of neurons layer 2 | 5 to 20 | 5 | 15 |

*Note.* Parameters without information on the increment had a finite range.

# Table S4. Sensitivity, specificity, PPP, and NPP at different thresholds

| **Threshold** | **Sensitivity** | **Specificity** | **PPP** | **NPP** |
| --- | --- | --- | --- | --- |
| 0.13 | 0.9465 | 0.2057 | 0.0637 | 0.9854 |
| 0.20 | 0.9256 | 0.2621 | 0.0668 | 0.9840 |
| 0.25 | 0.8903 | 0.3538 | 0.0729 | 0.9826 |
| 0.30 | 0.8449 | 0.4576 | 0.0817 | 0.9810 |
| 0.34 | 0.8015 | 0.5207 | 0.0872 | 0.9787 |
| 0.40 | 0.7569 | 0.5947 | 0.0964 | 0.9772 |
| 0.45 | 0.7166 | 0.6503 | 0.1048 | 0.9757 |
| 0.50 | 0.6735 | 0.6976 | 0.1128 | 0.9740 |
| 0.55 | 0.6324 | 0.7464 | 0.1246 | 0.9726 |
| 0.60 | 0.5308 | 0.8293 | 0.1508 | 0.9687 |
| 0.65 | 0.4510 | 0.8825 | 0.1797 | 0.9657 |
| 0.70 | 0.4098 | 0.9083 | 0.2033 | 0.9642 |
| 0.75 | 0.3676 | 0.9309 | 0.2331 | 0.9627 |
| 0.783 | 0.3253 | 0.9500 | 0.2707 | 0.9610 |
| 0.80 | 0.2811 | 0.9635 | 0.3056 | 0.9591 |
| 0.85 | 0.1900 | 0.9823 | 0.3801 | 0.9550 |
| 0.90 | 0.0900 | 0.9938 | 0.4514 | 0.9503 |

*Note.* PPP=positive predictive power; NPP=negative predictive power.

# Fig. S1. Training history (loss and AUC) for the DNN.


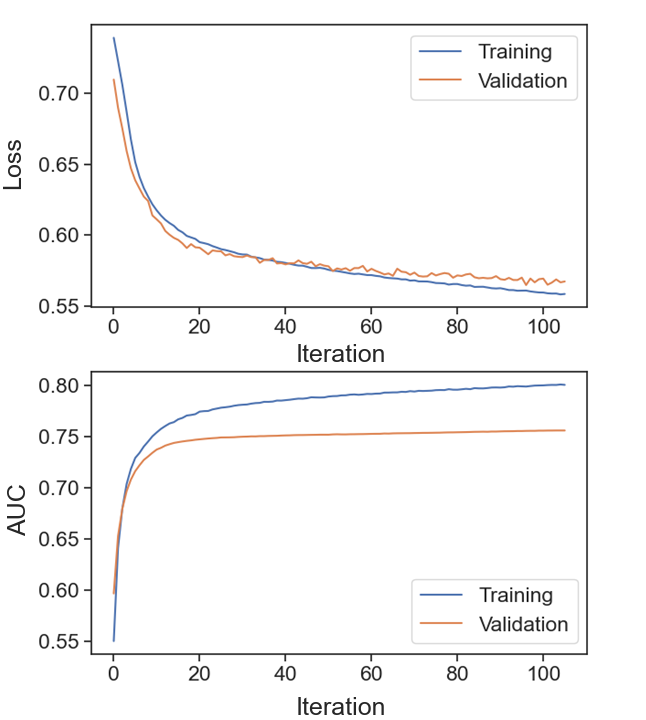


# Fig. S2. Learning curves for the DNN models showing model accuracy.


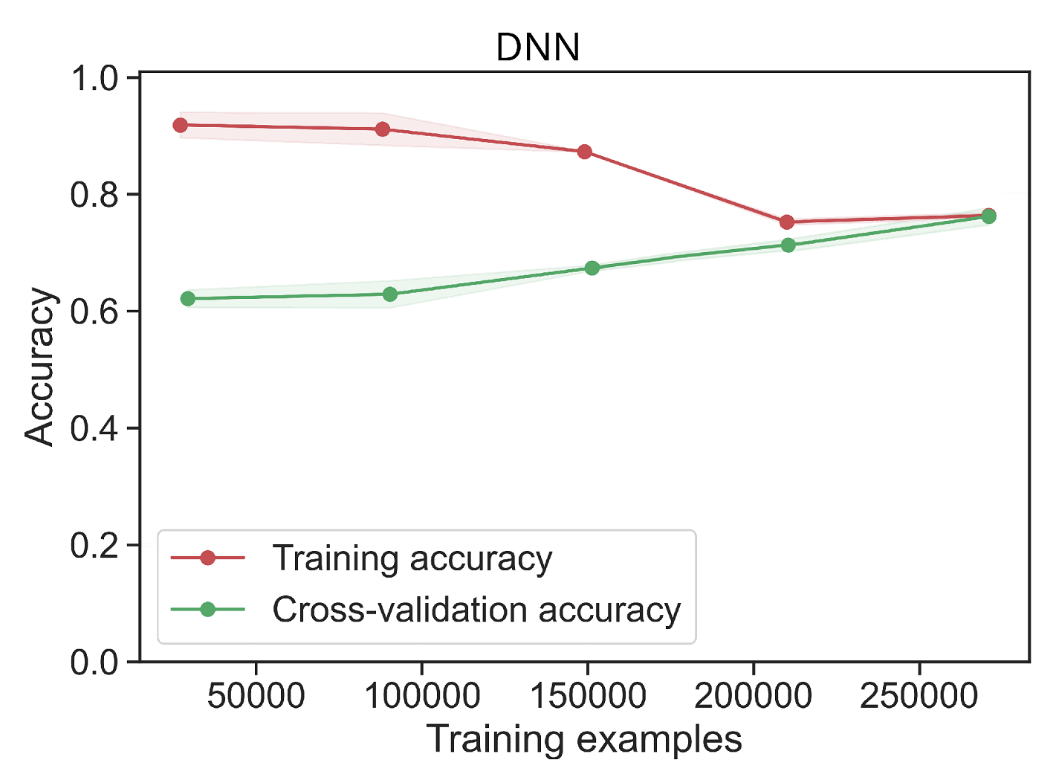


# Fig. S3. SHAP values explaining the predicted probability for ADHD of three different individuals.


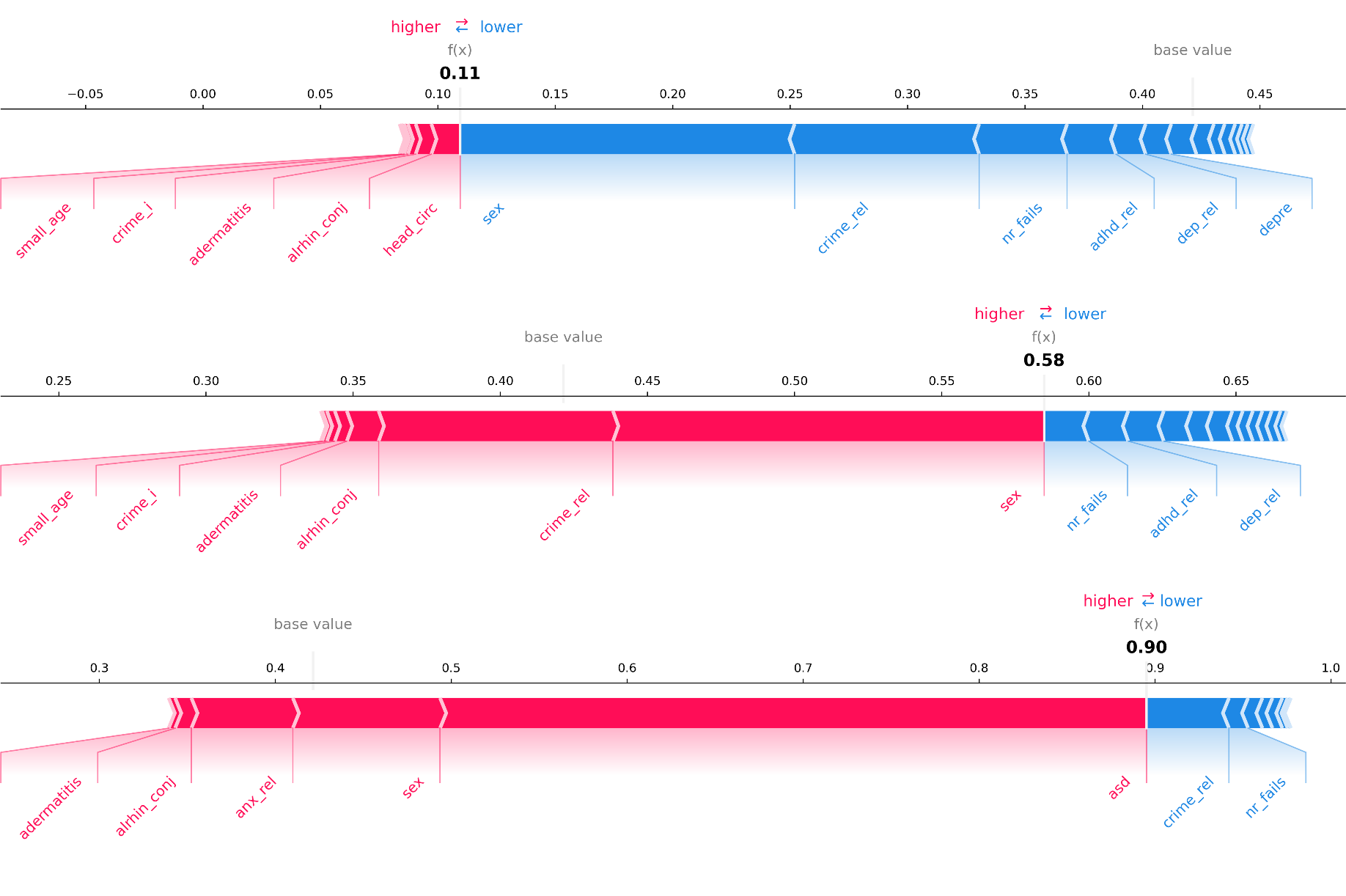


# STROBE Statement

Checklist of items that should be included in reports of observational studies

| **Section/Topic** | Item No | Recommendation | Reported on Page No | |
| --- | --- | --- | --- | --- |
| **Title and abstract** | 1 | (*a*) Indicate the study’s design with a commonly used term in the title or the abstract | 1 | |
|  |  | (*b*) Provide in the abstract an informative and balanced summary of what was done and what was found | 1 | |
| Introduction | | | |  |
| Background/rationale | 2 | Explain the scientific background and rationale for the investigation being reported | 3-4 | |
| Objectives | 3 | State specific objectives, including any prespecified hypotheses | 3-4 | |
| Methods | | | |  |
| Study design | 4 | Present key elements of study design early in the paper | 4 | |
| Setting | 5 | Describe the setting, locations, and relevant dates, including periods of recruitment, exposure, follow-up, and data collection | 4-6 | |
| Participants | 6 | (*a*) *Cohort study*—Give the eligibility criteria, and the sources and methods of selection of participants. Describe methods of follow-up  *Case-control study*—Give the eligibility criteria, and the sources and methods of case ascertainment and control selection. Give the rationale for the choice of cases and controls  *Cross-sectional study*—Give the eligibility criteria, and the sources and methods of selection of participants | 4-6 | |
|  |  | (*b*) *Cohort study*—For matched studies, give matching criteria and number of exposed and unexposed  *Case-control study*—For matched studies, give matching criteria and the number of controls per case |  | |
| Variables | 7 | Clearly define all outcomes, exposures, predictors, potential confounders, and effect modifiers. Give diagnostic criteria, if applicable | 4-6 | |
| Data sources/measurement | 8* | For each variable of interest, give sources of data and details of methods of assessment (measurement). Describe comparability of assessment methods if there is more than one group | 4-6 | |
| Bias | 9 | Describe any efforts to address potential sources of bias | 6-7 | |
| Study size | 10 | Explain how the study size was arrived at | 8 | |
| Quantitative variables | 11 | Explain how quantitative variables were handled in the analyses. If applicable, describe which groupings were chosen and why | 6 | |
| Statistical methods | 12 | (*a*) Describe all statistical methods, including those used to control for confounding | 6-8 | |
|  |  | (*b*) Describe any methods used to examine subgroups and interactions | 6-8 | |
|  |  | (*c*) Explain how missing data were addressed | 6-8 | |
|  |  | (*d*) *Cohort study*—If applicable, explain how loss to follow-up was addressed  *Case-control study*—If applicable, explain how matching of cases and controls was addressed  *Cross-sectional study*—If applicable, describe analytical methods taking account of sampling strategy | 5 | |
|  |  | (*e*) Describe any sensitivity analyses |  | |

| **Section/Topic** | Item No | Recommendation | Reported on Page No |
| --- | --- | --- | --- |
| Results | | | |
| Participants | 13* | (a) Report numbers of individuals at each stage of study—eg numbers potentially eligible, examined for eligibility, confirmed eligible, included in the study, completing follow-up, and analysed | 8 |
|  |  | (b) Give reasons for non-participation at each stage |  |
|  |  | (c) Consider use of a flow diagram |  |
| Descriptive data | 14* | (a) Give characteristics of study participants (eg demographic, clinical, social) and information on exposures and potential confounders | 6, Table S1 |
|  |  | (b) Indicate number of participants with missing data for each variable of interest | Table S1 |
|  |  | (c) *Cohort study*—Summarise follow-up time (eg, average and total amount) |  |
| n | 15* | *Cohort study*—Report numbers of outcome events or summary measures over time | 8 |
|  |  | *Case-control study—*Report numbers in each exposure category, or summary measures of exposure |  |
|  |  | *Cross-sectional study—*Report numbers of outcome events or summary measures |  |
| Main results | 16 | (*a*) Give unadjusted estimates and, if applicable, confounder-adjusted estimates and their precision (eg, 95% confidence interval). Make clear which confounders were adjusted for and why they were included | 9-10 |
|  |  | (*b*) Report category boundaries when continuous variables were categorized |  |
|  |  | (*c*) If relevant, consider translating estimates of relative risk into absolute risk for a meaningful time period |  |
| Other analyses | 17 | Report other analyses done—eg analyses of subgroups and interactions, and sensitivity analyses |  |
| Discussion | | | |
| Key results | 18 | Summarise key results with reference to study objectives | 11-14 |
| Limitations | 19 | Discuss limitations of the study, taking into account sources of potential bias or imprecision. Discuss both direction and magnitude of any potential bias | 13 |
| Interpretation | 20 | Give a cautious overall interpretation of results considering objectives, limitations, multiplicity of analyses, results from similar studies, and other relevant evidence | 13 |
| Generalisability | 21 | Discuss the generalisability (external validity) of the study results | 13 |
| Other Information | | | |
| Funding | 22 | Give the source of funding and the role of the funders for the present study and, if applicable, for the original study on which the present article is based | 15 |

**Give information separately for cases and controls in case-control studies and, if applicable, for exposed and unexposed groups in cohort and cross-sectional studies.*

**Note:** An Explanation and Elaboration article discusses each checklist item and gives methodological background and published examples of transparent reporting. The STROBE checklist is best used in conjunction with this article (freely available on the Web sites of PLoS Medicine at http://www.plosmedicine.org/, Annals of Internal Medicine at http://www.annals.org/, and Epidemiology at http://www.epidem.com/). Information on the STROBE Initiative is available at www.strobe-statement.org.

# Guidelines for Reporting Machine Learning Investigations in Neuropsychiatry (GREMLIN)

| **Section** | **Item  #** | **Checklist item** | **Explanation** | **Done** |
| --- | --- | --- | --- | --- |
| **TITLE** | | | |  |
| Title | 1 | Identify the use of machine learning for predictive or diagnostic purposes. | This will allow more efficient database searching and identification of relevant studies. | 🗸 |
| **ABSTRACT** | | | |  |
| Article summary | 2 | Structured summary including background, aims, source data, model performance and conclusion. | The structured summary provides sufficient information for readers to grasp the range and main aspects of the study. | 🗸 |
| **INTRODUCTION** | | | |  |
| Background | 3 | Describe the current knowledgebase and how learning algorithms may lead to improved patient care or mechanistic insights. | Enable the reader to follow the process of developing the research question; from what is known and clinically utilised in psychiatry, to the application and anticipated benefits of machine learning algorithms. | 🗸 |
| Aims | 4 | Describe the purpose of ML in terms of hypothesis testing or prediction. | Model interpretability is critical for hypotheses testing. Model interpretability is not critical for prediction purposes. | 🗸 |
|  | 5 | Predictive modelling: prognostic or diagnostic? | Predictive modelling may be prognostic (time to an event) or diagnostic (correctly identifying that a condition already exists). | 🗸 |
|  | 6 | Frame the research question with respect to PICOS | PICOS: Population, intervention, comparator, outcome, study design.  More information regarding PICOS can be found in most institutional library guides. | 🗸 |
|  |  |  |  |  |
| **METHODS** | | | |  |
| Study design | 7 | Provide a full description, or reference to a full description of the population upon which modelling will occur; facility, screening dates and numbers, inclusion/exclusion criteria, database/study name, study duration. | Is the patient population from a prospective or retrospective study? For retrospective studies (where recruitment and data collection have already been performed), the reader must be able to gauge the fitness of the study for the current application. | 🗸 |
|  | 8 | Include ethics statement and approval number |  | 🗸 |
| Prediction environment | 9 | Describe the outcome measurement. | Outcome measurement(s) may include prediction per patient or per event. | 🗸 |
|  | 10 | Classification, regression or survival prediction | Classification is the prediction of a categorical label (such as good outcome or poor outcome).  Regression is the prediction of a quantity or continuous variable (such as score for depression).  Survival prediction is time to event and requires identification of the event (such as remission) and time measurement (such as weeks). | 🗸 |
|  | 11 | Training and validation environments | Describe the process for model training and validation. This must include how data was partitioned. For example, how many patient samples contributed data for training? Was cross-validation used? How many folds? | 🗸 |
|  | 12 |  | Describe the process for hyperparameter optimisation. What parameters were tuned (eg Gamma, C), how were they tuned (eg grid search, random search or Bayes optimisation) and what ranges were used? Which optimisation criterion was used (eg prognostic summary index). Was model complexity accounted for? | 🗸 |
|  | 13 | Model testing | Model testing must be performed on new/unseen data. Describe the metrics to be used for assessment of model performance. For example sensitivity, specificity, positive predictive value, negative predictive value, diagnostic odds ratio, Area Under the (receiver operating characteristic) Curve (AUC). | 🗸 |
|  | 14 | Data leakage | Data leakage may occur in relation to outcomes or validation and may lead to inflated prediction performance. The use of cross-validation, holding back data (unseen) for later validation and the use of pipeline architecture can reduce bias in model performance. Furthermore, data transformations should be performed separately on data partitions and not the entire data set to avoid leaking feature distribution information between folds. | 🗸 |
|  | 15 | Prediction success | How will the model be determined as adequate for its purpose and how does it compare to other models? Model adequacy may include measures of discrimination and calibration. What statistical procedures were used for model comparison?  Global measures of diagnostic accuracy, such as Area Under Receiver Operating Characteristic Curve (AUC) allow for model comparison. | 🗸 |
|  | 16 | Overfitting | Overfitting occurs when learning on the training data set is excessive and irrelevant or noisy features are included in selection leading to inflated model performance on training data but poor performance on unseen data. This can be overcome with k-fold cross validation and hold back validation data. | 🗸 |
| Data acquisition and pre-processing | 17 | Present characteristics of the dataset | Provide relevant summary statistics for the dataset and particular information for the distribution or ratios of response variables. | 🗸 |
|  | 18 | Explain the process for handling missing values, class imbalance, covariates and outliers | Clearly state where data were discarded or imputed (for example variables with >=20% missing values excluded). Manage class imbalance at data or algorithm level, or a combination of these. | 🗸 |
|  | 19 | Check data for perfect separators | The data should be checked for uncommon values for categorical variables that may lead to overfitting. This can be overcome by removing the variable from selected features and assessing the impact. | 🗸 |
|  | 20 | Data scaling | What method was used for data scaling?  For example Z-score normalisation or Min-Max scaling (0-1). | 🗸 |
| Outcomes, variables | 21 | Predictor variables | Describe predictor variables. | 🗸 |
|  | 22 | Generalisability | Identify how generalisability will be addressed. For example, cross-validation, leave site out validation. | 🗸 |
|  | 23 | Code/algorithm | Where in-house code is developed, it should be made available (for example deposited on Github), with appropriate commentary, to allow inspection.  Where a code package was used (for example R package e1071, Python package Scikit-learn and NeuroMiner) this should be stated. | 🗸 |
| **RESULTS** | | | |  |
| Model performance | 24 | Describe model performance | Model performance to be evaluated based on quality metrics described in methods.  Additionally, standard reporting of ML results should include confidence intervals, AUC and Balanced Accuracies (BAC), to allow for comparison of model performance with other published studies. | 🗸 |
|  | 25 | How does the final model compare to other predictive tools? | Refer to model selection criteria as outlined in methods. | 🗸 |
|  | 26 | Bias and variance assessment | Prediction errors can be assessed with perturbation resampling, bootstrap resampling etc. | 🗸 |
|  | 27 | Model output interpretation | Where possible, report the variables used for prediction of outcome(s).  Report population subsets that were challenging, or easy, to predict. | 🗸 |
| **DISCUSSION** | | | |  |
| Clinical implementation | 28 | Describe how the model may be harnessed for improved patient care | Describe how the patient stands to benefit from; this may include savings in time, finances, side-effects.  Describe the most appropriate setting for application of the model. | 🗸 |
| Model limitations | 29 | Data format | State if there are any particular data format requirements, especially those that may hinder widespread use of the model in an appropriate setting. | 🗸 |
|  | 30 | Justify choice of ML method | This may be related to purpose (eg interpretability), data characteristics (modality, quantity and quality), computational resources. | 🗸 |
|  | 31 | Discuss potential bias in data sampling |  |  |
|  | 32 | Discuss generalisability of the model | Does the model have applicability to other sample collection sites and particularly sites with different outcome distributions? Leave group out cross-validation can address site/group effects. | 🗸 |
|  | | | |  |

# Code availability and use

The code used to train each model is available at https://github.com/kmlstyle/ADHD-DNN together with the deep neural network weights to load and use with any data. The code is separated into two files, one for the machine learning models and another for the deep neural network model. Each model expects an array with 22 features and the order of each feature can be found within the Python code. The Public Access to Information and Secrecy Act in Sweden prohibits us from making individual level data publicly available. Researchers who are interested in replicating our work can apply for individual level data at Statistics Sweden: www.scb.se/en/services/guidance-for-researchers-and-universities/.
